# Quality by Design Optimization of Cold Sonochemical Synthesis of Zidovudine-Lamivudine Nanosuspensions

**DOI:** 10.3390/pharmaceutics12040367

**Published:** 2020-04-17

**Authors:** Bwalya A. Witika, Vincent J. Smith, Roderick B. Walker

**Affiliations:** 1 Division of Pharmaceutics, Faculty of Pharmacy, Rhodes University, Makhanda 6140, South Africa; bwawitss@gmail.com; 2 Department of Chemistry, Faculty of Science, Rhodes University, Makhanda 6140, South Africa; v.smith@ru.ac.za

**Keywords:** nano co-crystals, design of experiments, Quality by Design, crystal engineering, sonochemistry, HIV/AIDS, lamivudine, zidovudine

## Abstract

Lamivudine (3TC) and zidovudine (AZT) are antiviral agents used to manage HIV/AIDS infection. The compounds require frequent dosing, exhibit unpredictable bioavailability and a side effect profile that includes hepato- and haema-toxicity. A novel pseudo one-solvent bottom-up approach and Design of Experiments using sodium dodecyl sulphate (SDS) and α-tocopheryl polyethylene glycol succinate 1000 (TPGS 1000) to electrosterically stablize the nano co-crystals was used to develop, produce and optimize 3TC and AZT nano co-crystals. Equimolar solutions of 3TC in surfactant dissolved in de-ionised water and AZT in methanol were rapidly injected into a vessel and sonicated at 4 °C. The resultant suspensions were characterized using a Zetasizer and the particle size, polydispersity index and Zeta potential determined. Optimization of the nanosuspensions was conducted using a Central Composite Design to produce nano co-crystals with specific identified and desirable Critical Quality Attributes including particle size (PS) < 1000 nm, polydispersity index (PDI) < 0.500 and Zeta potential (ZP) < −30mV. Further characterization was undertaken using Fourier Transform infrared spectroscopy, energy dispersive X-ray spectroscopy, differential scanning calorimetry, powder X-ray diffraction and transmission electron microscopy. In vitro cytotoxicity studies revealed that the optimized nano co-crystals reduced the toxicity of AZT and 3TC to HeLa cells.

## 1. Introduction

Since the beginning of the HIV epidemic in the 1980s, 74.9 million people have been infected with the virus. In the same time period, an approximate total of 32 million people have died from AIDS-related illnesses [1].

According to a report by UNAIDS, approximately 37.9 million people globally were living with HIV at the end of 2018. Of these, 23.3 million were accessing antiretroviral therapy (ART). In addition, 1.7 million new HIV infections were reported in 2018. HIV/AIDS also accounted for 770,000 deaths from AIDS-related illnesses [1].

Co-crystals incorporate pharmaceutically acceptable guest molecules into a crystal together with an active pharmaceutical ingredient (API). Co-crystals have gained attention as alternate solid forms for drug development [2]. For pharmaceutical applications, co-crystallization is highly promising for tailoring the properties of Active Pharmaceutical Ingredients (API) and co-former couples to improve dissolution kinetics, the rate and extent of bioavailability, and the stability of compounds [3,4,5,6].

Multi-API co-crystals, which remain relatively unexplored solid forms of API, have potential relevance in the context of combination therapy for drug and product development [7]. AZT and 3TC are antiviral agents used in combination for the suppression and prevention of HIV/AIDS [3,8,9,10,11]. The 3TC·AZT·H_2_O co-crystal has been synthesized by neat grinding, liquid-assisted grinding and solvent evaporation [12]. The co-crystal has the potential for co-delivery of both molecules.

Co-crystals can be produced in the nanometre range using top-down or bottom-up manufacturing approaches. The production of nanocrystals and nano co-crystals using bottom-up techniques generally involve the use of a solvent system(s) added to an anti-solvent system in the presence of ionic and/or non-ionic stabilisers. The mixing of drug solution(s) and anti-solvent is generally achieved with conventional mixing equipment such as magnetic and/or overhead stirrers fitted with an agitator blade [13]. In order to promote nucleation, sonic waves are introduced using a process now called, sonoprecipitation [13,14]. High-energy mechanical forces are involved when using top-down approaches, which are achieved using media milling (MM) (NanoCrystals^®^) or High Pressure Homogenization (IDD-P^®^, DissoCubes^®^ and Nanopure^®^) to comminute large crystals [15,16].

The use of surfactants and polymers such as Tween ^®^, Span ^®^, hydroxypropyl methylcellulose (HPMC), pyrrolidone K30 and polyvinyl pyrrolidone as stabilizers has been explored in the synthesis of nanocrystals and nano co-crystals (NCC) [17,18,19,20,21,22,23,24,25].

Macrophages are carriers of HIV in humans [26,27,28] and targeting these cells by use of nanotechnological approaches has been proposed as an ideal option for more efficient treatment of HIV and halting the progression to AIDS [29,30,31,32]. Nanocrystals and nano co-crystals offer a potential route for targeting macrophages. Following their administration, nanocrystals and nano co-crystals are engulfed by phagocytic cells such as macrophages after recognition as foreign bodies. In phagocytic cells, nano-dimensional crystals dissolve slowly in phagolysosomes. Consequently, any payload might pass through phagolysosomal membranes and reach the cytoplasm of cells, following which diffusion from the cell down a concentration gradient occurs [15].

Cold-sonochemical co-crystallization as a process has been used successfully for the manufacture of nano co-crystals [14,33,34]. One-solvent systems involve dissolving the components in a single solvent and injecting the solution into an anti-solvent while applying ultrasonic energy [33], whereas in two-solvent systems, the components are dissolved in separate vehicles and injected into an anti-solvent. Preliminary studies suggested that neither of these approaches were suitable due to differences in the solubility of 3TC and AZT. Consequently, we developed a pseudo one-solvent system approach in which the components were dissolved in separate solvents that acted as anti-solvents for each other in situ [25].

The pseudo one-solvent cold-sonochemical approach [25] was used to synthesize and optimize co-crystals in the nanometer range with a specific Zeta potential that could potentially exploit the advantages of nanometer drug delivery systems for targeted drug delivery [35,36] as well as those of co-crystalline drug delivery systems such as enhanced solubility [37,38] and for combination therapy [39]. We have previously demonstrated the preparation of self-assembled electrosterically (both sterically and electrostatically) stabilized nano co-crystals using SDS as an electrostatic stabilizer and TPGS 1000 as a steric stabilizer using an appropriate anti-solvent [25].

A Design of Experiments (DoE) approach, specifically Central Composite Design (CCD) with the aid of Response Surface Methodology (RSM) was used to optimize formulation variables for the synthesis of 3TC and AZT nano co-crystals using cold-sonoprecipitation. Preliminary studies revealed that two independent factors viz., SDS and TPGS 1000 concentration were important.

The main purpose of these studies was to develop and optimize, using the principles of Quality by Design (QbD), a surface-modified nano co-crystal formulation, using pseudo one-solvent bottom-up cold-sonoprecipitation. The NCC will have the potential to deliver 3TC and AZT to target reservoirs of HIV in different tissues, with the potential of improving the side effect profile of each API by reducing the dose. The surfactants, TPGS 1000 and SDS were identified and reported previously [25] and the optimization of the formulation and cytotoxicity of the nano co-crystal is reported herein.

## 2. Materials and Methods

### 2.1. Materials

AZT and 3TC were purchased from China Skyrun Co. Ltd. (Taizhou, China). SDS and TPGS 1000 were purchased from Merck (Johannesburg, South Africa). HPLC-grade water was prepared by reverse osmosis using a RephiLe^®^ Direct-Pure UP and RO water system Microsep^®^ (Johannesburg, South Africa) fitted with a RephiDuo^®^ H PAK de-ionization cartridge and a RephiDuo^®^ PAK polishing cartridge. The water was filtered through a 0.22 µm PES high-flux capsule filter Microsep^®^ (Johannesburg, South Africa) prior to use. HPLC-grade methanol (MeOH) was purchased from Honeywell Burdick and Jackson™ (Anatech Instruments, Johannesburg, South Africa).

### 2.2. Methods

#### 2.2.1. Nano Co-Crystal Synthesis

NCC were synthesized using a cold-sonoprecipitation pseudo one-solvent method as previously described [25]. The batch size was approximately 1 g, specifically 534 mg of AZT and 458 mg of 3TC equivalent to 2 mmol of each API. 3TC was dissolved in 7 mL water and AZT in 6 mL MeOH. The surfactants were added to 3TC/water solution in concentrations as required by the CCD. The solutions were rapidly injected into a pre-cooled conical flask, and then incubated at 4 °C ± 2 °C in an ice bath. A sonication output of 50 kHz ± 6 kHz was applied to the solution for 20 min using a Branson^®^ 8510E-MT ultrasonic bath (Branson Ultrasonics Corp., Danbury, CT, USA).

#### 2.2.2. Nano Co-Crystal Optimization

A CCD design generated using version 11 Design Expert^®^ software (Stat-Ease Inc., Minneapolis, MN, USA) was selected for the optimization of process variables for the synthesis of NCC using reduced-temperature sonoprecipitation. Preliminary studies revealed that two independent factors viz., SDS and TPGS 1000 concentration were critical [25]. The PS, PDI and ZP were monitored. The experiments conducted for the CCD are listed in Table 1. All experiments were performed in triplicate for each run using the levels suggested by the CCD, which also includes repeated runs at specific levels. Furthermore, the runs are conducted in a randomised manner to minimize any potential bias.

#### 2.2.3. Particle Size Analysis

The mean PS and PDI of NCC was determined using a Nano-ZS 90 Zetasizer (Malvern Instruments, Worcestershire, UK) with the instrument set in Photon Correlation Spectroscopy (PCS) mode. Approximately 30 μL of an aqueous dispersion of NCC was diluted with 10 mL HPLC-grade water prior to analysis. The sample was placed into a 10 × 10 × 45 mm polystyrene cell and all measurements were performed in replicate (*n* = 6) at 25 °C using a standard 4 mW laser set at 633 nm at a scattering angle of 90°. Analysis of PCS data was undertaken using Mie theory with real and imaginary refractive indices set at 1.456 and 0.01.

#### 2.2.4. Zeta Potential

The ZP of NCC was measured using a Nano-ZS 90 Zetasizer (Malvern Instruments, Worcestershire, UK) set in Laser Doppler Anemometry (LDA) mode (*n* = 6) at a wavelength of 633 nm. The samples were prepared for analysis as described in Section 2.2.2 and placed into folded polystyrene capillary cells prior to analysis.

#### 2.2.5. Formulation Optimization

Numerical optimization was undertaken using version 11 Design Expert^®^ software (Stat-Ease Inc.) with the aim of identifying an optimum formulation composition and associated formulation parameters that would ensure the production of nanosuspension formulations of optimum stability with the potential to target reservoirs of HIV in the body. Although different methods are used to achieve optimization of process and formulation parameters, the use of a numerical optimization is a comprehensive and effective approach for any continuous optimization process [40]. Numerical optimization locates a point in space that maximises the desirability function while modifying the characteristics of a target by adjusting the importance of that target [41]. The summary of formulation parameters used for the optimization of nano co-crystals are listed in Table 2.

### 2.3. Characterization of Optimized Nano Co-Crystal (OPT-NCC)

#### 2.3.1. Differential Scanning Calorimetry

Approximately 4 mg of filtered and air-dried OPT-NCC (to constant mass) was placed into an aluminum pan and sealed. The pan was then placed directly into the furnace of a DSC 6000 PerkinElmer Differential Scanning Calorimeter (Waltham, MA, USA) and the data generated was analyzed using version 11 Pyris^™^ Manager Software (PerkinElmer). The temperature of the DSC was monitored with a computer and a controlled heating rate of 10 K/min was used for the analysis over the temperature range 30–150 °C. All DSC analyses were conducted in triplicate (*n* = 3) under a nitrogen atmosphere purged at a flow rate of 20 mL/min and the thermogram for the micro co-crystal (uncoated) was used as a reference.

#### 2.3.2. Energy-Dispersive X-ray Spectroscopy Scanning Electron Microscopy

Elemental analysis was performed using a Vega^®^ Scanning Electron Microscope (TESCAN, Brno, Czechia) fitted with an INCA PENTA FET. Approximately 1 mg of the OPT-NCC was dusted onto a graphite plate and the sample irradiated at an accelerated voltage of 20 kV (*n* = 3).

#### 2.3.3. FTIR Spectroscopy

The IR absorption spectrum of the OPT-NCC was generated using a Model 100 Spectrum FTIR ATR Spectrophotometer (PerkinElmer) and analyzed using version 4.00 Peak^®^ Spectroscopy software (Operant LLC, Burke, VA, USA). Approximately 5 mg powder was placed onto a diamond crystal and analyzed over the wavenumber range 4000–650 cm^−1^ at a rate of 4 cm^−1^ in replicate (*n* = 5) and the spectrum for the uncoated nano co-crystals was used for reference purposes.

#### 2.3.4. Powder X-ray Diffraction (PXRD)

X-ray powder diffraction patterns were collected using a Bruker D8 Discover diffractometer (Bruker, Billerica, MA, USA) equipped with a proportional counter, Cu-Kα radiation of λ = 1.5405 Å and a nickel filter. Samples were placed onto a silicon wafer slide. Generator settings were 30 kV with a current of 40 mA used for the measurement. Data were collected (*n* = 3) in the range 2θ = 10° to 50° at a scanning rate of 1.5° min^−1^ with a filter time-constant of 0.38 s per step and a slit width of 6.0 mm. The X-ray diffraction data were treated using evaluation curve fitting (Diffrac.Eva, version: V2.9.0.22, Bruker) software. Baseline correction was performed on each diffraction pattern by subtracting a spline function fitted to the curved background and the diffraction pattern of uncoated nano co-crystals was used for reference purposes.

#### 2.3.5. Transmission Electron Microscopy (TEM)

TEM was used to visualize the shape of the NCC in the original aqueous dispersion. A drop of the aqueous dispersion was placed onto a copper grid with a carbon film and excess liquid was removed using Whatman^®^ 110 filter paper (Whatman PLC, Maidstone, England), after which the sample was dried at room temperature (22 °C) for 24 h. The sample was visualised using a Zeiss Libra^®^ 120 TEM (Carl Zeiss AG, Munich, Germany).

#### 2.3.6. In Vitro Cytotoxicity Studies

HeLa (human cervix adenocarcinoma cells) (Cellonex) were cultured in Dulbecco’s Modified Eagle Medium (DMEM)-(Lonza) supplemented with 10% w/w fetal calf serum and antibiotics (penicillin/streptomycin/amphotericin B) at 37 °C in a 5% CO_2_ incubator. HeLa cells were transferred to 96-well plates at a cell density of 1 × 10^4^ cells per well in 150 µL culture medium and grown overnight. A single concentration of 50 µM of the test compound was incubated with the cells for an additional 48 h, and cell viability in the wells assessed by adding 20 µL 0.54 mM resazurin in PBS for an additional 2–4 h. The numbers of cells surviving drug treatment were determined by reading resorufin fluorescence (excitation 560 nm, emission 590 nm) with a SpectraMax^®^ M3 plate reader (Molecular Devices, San Jose, CA, USA). Fluorescence readings for the individual wells were converted to percent (%) cell viability relative to the average readings from untreated control wells. Plots of % cell viability vs. log(compound) were used to determine IC_50_ values by non-linear regression using version 5.02 GraphPad Prism (GraphPad Holdings LLC, La Jolla, CA, USA).

The compounds investigated were the optimized NCC, the individual API and a physical mixture of the API in stoichiometric ratios identical to those used to produce the NCC.

## 3. Results

### 3.1. Optimization of Electrosteric NCC

A summary of the input variables used to optimize the manufacture of NCC identified using the CCD are summarized in Table 3.

#### 3.1.1. Response Surface Quadratic Model for PS (Y_1_)

The ANOVA results for the response surface quadratic model for PS are listed in Table 4. The model F value was 26.82 indicating that the model was significant. The model F-value is used to ascertain the utility of a model that the data has been fitted to and determine whether the data is best fitted by the model. The F-value is explained and unexplained variability and the larger the F-value, the more useful the model [42]. The particle size was influenced by SDS and TPGS 1000 concentration and an increase in % w/v content of each resulted in a reduction in particle size.

The mean PS of the NCC fell between 304.1 and 859.4 nm. The impact of SDS on the size of NCC resulted in a large F-value of 83.23, indicating that this parameter had a significant impact on the size of NCC produced when compared to the other variable investigated. However, the TPGS 1000 content and the quadratic effect of the SDS concentration produced F-values of 29.29 and 36.65, respectively, indicating that these parameters had an intermediate, yet significant, impact on the resultant size of the NCC produced.

**Figure 1:**
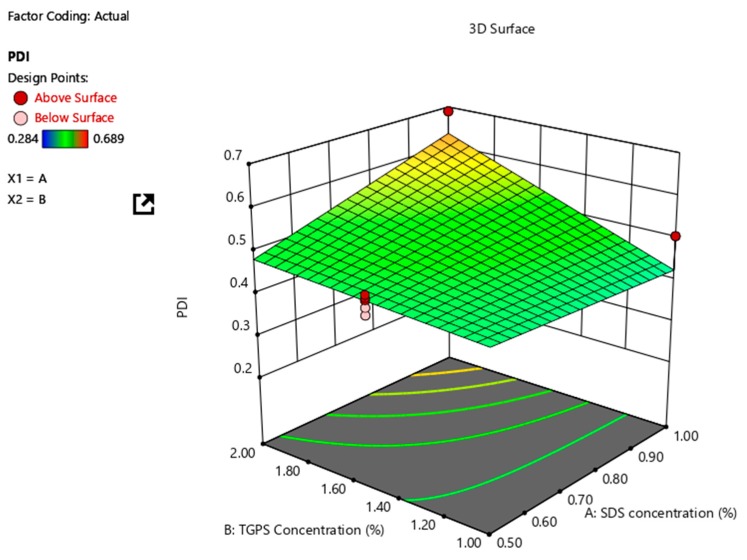
3D response surface plot depicting the impact of SDS and TPGS 1000 concentration on particle size of the NCC.

The three-dimensional (3D) response surface plot in which the impact of SDS and TPGS 1000 concentration on particle size is depicted in Figure 1. These data reveal that a synergistic relationship exists between the SDS and TPGS 1000 concentration on particle size. The surface plots reveal that relatively small NCC are produced when the amount of SDS used is high and TPGS 1000 content is constant. The same effect is observed when the SDS concentration is constant and TGPS 1000 concentration is increased. However, this effect is minimal when SDS concentrations are at a maximum of 1% w/v. These results are consistent with previous findings that reported a decrease in mean PS with increasing content of an electrostatic or steric surfactant [20,43,44,45]. The desired mean particle size can be produced by a manipulation of the combined effects of SDS and TPGS 1000 when producing NCC using this method.

#### 3.1.2. Response Surface Model for PDI (Y_2_)

The ANOVA results for the response surface linear model for PDI are listed in Table 5. The model F value was 6.37, indicating that the model was significant. Increasing the amount of SDS used increases the PDI.

**Figure 2:**
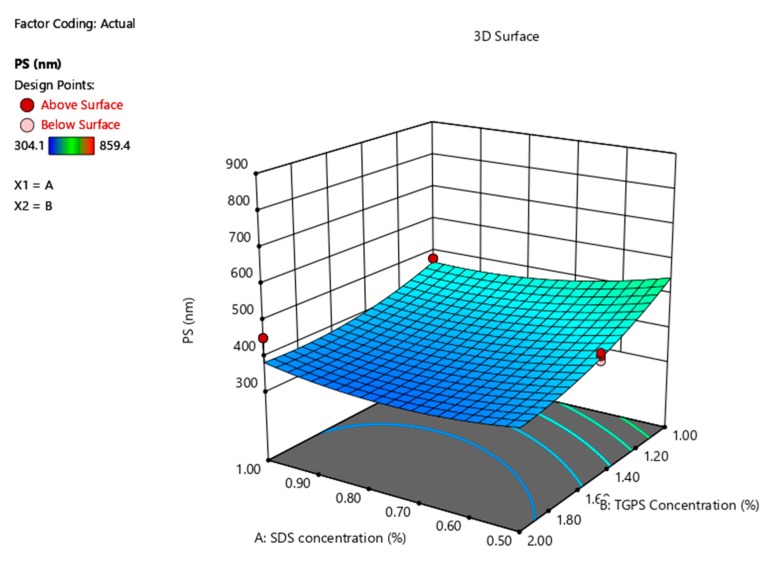
3D response surface plot depicting the impact of SDS and TPGS 1000 concentration on the PDI of the NCC.

The 3D response surface plot in Figure 2 depicts the impact of SDS concentration on PDI and that a linear correlation exists. A reduction of TPGS 1000 concentration appears to increase the PDI for the NCC when the SDS concentration is kept constant, but only marginally.

#### 3.1.3. Response Surface Model for ZP (Y_3_)

The ANOVA results for the response surface quadratic model for ZP are listed in Table 6. The model F value was 17.56, indicating that the model was significant. Increasing the amount of SDS used results in a decrease in ZP.

**Figure 3:**
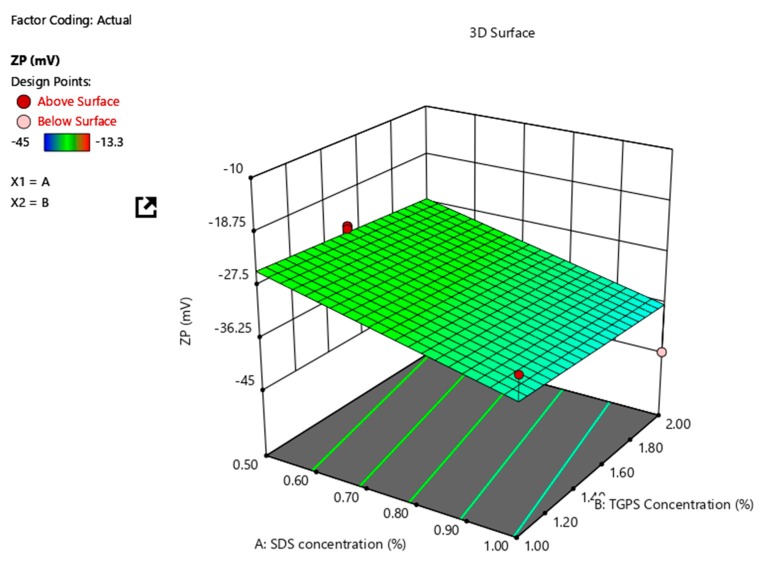
3D response surface plot depicting the impact of SDS and TPGS 1000 concentration on ZP of the NCC.

The 3D response surface plot in Figure 3 depicts the impact of SDS concentration on the ZP and that a linear correlation exists between SDS content and ZP. The concentration of TPGS 1000 appears to have an insignificant impact on the ZP of the NCC. The effect of SDS on ZP is consistent with that previously reported for SDS-stabilized nanosuspensions [46,47,48,49,50].

#### 3.1.4. Formulation Optimization

The desired target level for each input variable and associated responses PS, PDI and ZP are summarised in Table 7. The desirability of the model generated was 1.000, indicating that the optimum conditions were located in the desirability zone. The optimum formulation composition for the manufacture OPT-NCC and is summarised in Table 7.

The responses generated using the NCC formulation developed and manufactured according to the optimum composition are summarised in Table 8, in addition to the experimental and predicted responses with the corresponding percent prediction error.

### 3.2. Characterization of OPT-NCC

#### 3.2.1. Differential Scanning Calorimetry

**Figure 4:**
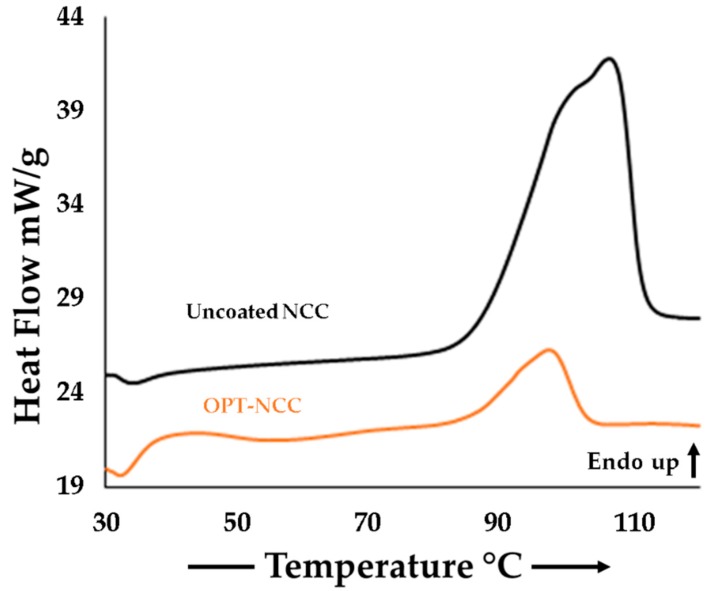
DSC thermograms depicting the melting endotherm for the uncoated (black) and OPT-NCC (orange).

DSC was used to investigate whether polymorphic changes had occurred when manufacturing the NCC and the resultant thermogram is depicted in Figure 4. The thermogram revealed a melting endotherm for the OPT-NCC with a T_peak_ at 94.3 °C and for the uncoated NCC at T_peak_ at 103.1 °C. The reduction in melting point is likely due to particle size reduction [51,52,53]. The NCC has a narrow melting endotherm, indicating that crystallinity is retained during the production process. Tabulated melting point data are reported in Table S1 and the thermograms for the components and the NCC are depicted in Figure S1 in the Supplementary Information.

#### 3.2.2. SEM-EDX

**Figure 5:**
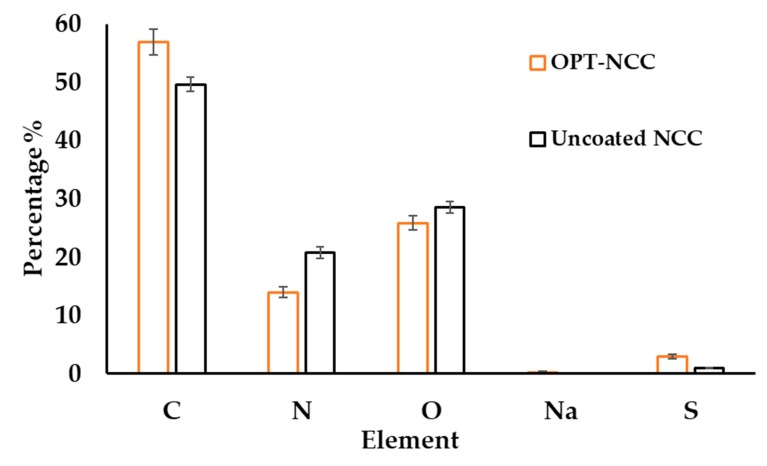
Elemental analysis of uncoated (black) and OPT-NCC (orange).

Elemental analysis indicates the presence of elemental sodium in SDS/TPGS stabilized NCC. The OPT-NCC have lower oxygen content when compared to uncoated NCC and the overall weight ratio is lower due to the presence of elemental sodium and high sulphur content from the combination of stabilizers used. A summary of the EDX measurements is listed in Table 9 and depicted in Figure 5.

#### 3.2.3. FTIR Spectroscopy

**Figure 6:**
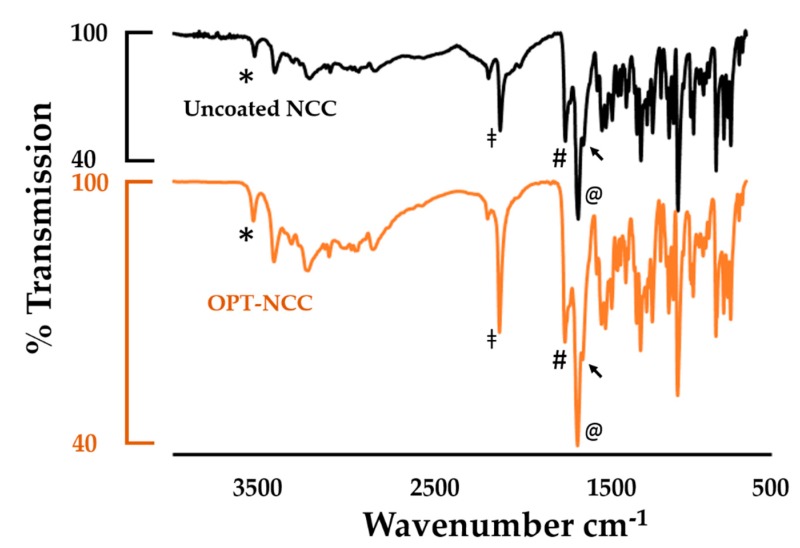
FTIR absorption spectra of the uncoated NCC (black) and the OPT-NCC (orange).

The FTIR spectra depicted in Figure 6 show peaks at 3530 cm^−1^ (*), which are characteristic of the hydrogen-bonded water in the crystal for both coated and uncoated nano co-crystals, [54,55]. The stretching band at 1635 cm^−1^ (@) is due to the carbonyl moiety (O=C–NR_2_) and is characteristic for 3TC and AZT. It partially overlaps with the N–H (bending) band at 1607 cm^−1^ (→). The stretching vibration of the imine group (R_2_-C=NR) is observed at 1648 cm^−1^ (#). Characteristic bands for AZT are observed at 2170 cm^−1^ (ǂ) and 1652 cm^−1^, due to –N_3_ and –N–H stretching vibrations. A comparison of the wavenumbers for the co-crystal and the OPT-NCC are reported and plotted in Table S2 and Figure S2 respectively and the spectra for the NCC, AZT and 3TC are depicted in Figure S3 in the Supplementary Information.

#### 3.2.4. PXRD

**Figure 7:**
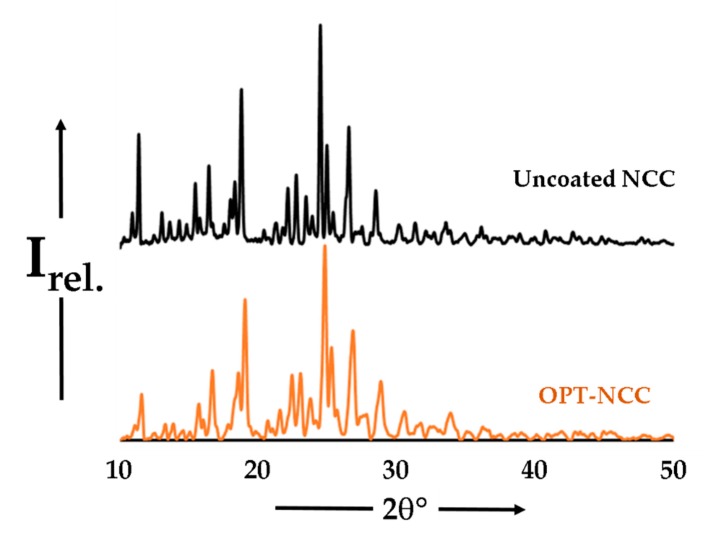
PXRD diffractograms for the uncoated (black) and OPT-NCC (orange).

The diffractograms depicted in Figure 7 are virtually indistinguishable, with a near one-to-one agreement in peak position, providing convincing evidence that they are the same phase or crystal structure [56,57]. A comparison of the calculated PXRD profile for the co-crystal and the experimental OPT-NCC profile is depicted in Figure S4 in the Supplementary Information. 

#### 3.2.5. Transmission Electron Microscopy (TEM)

**Figure 8:**
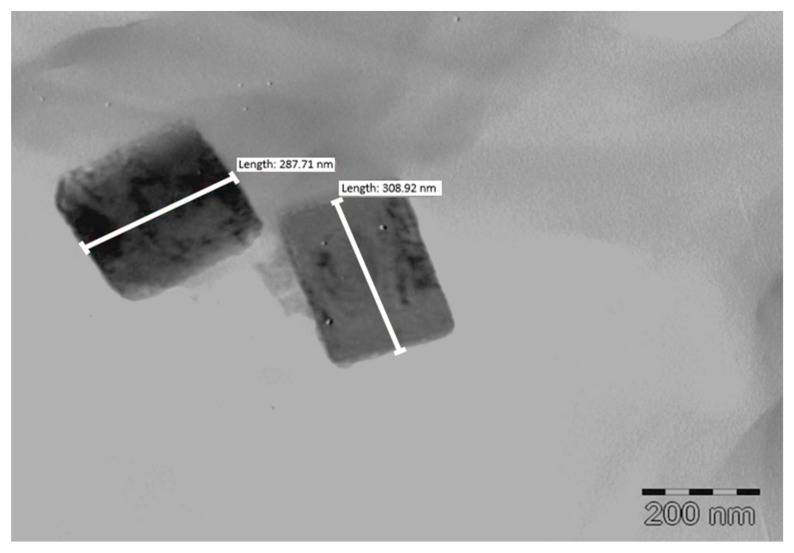
TEM micrograph of prismatic OPT-NCC.

The TEM micrograph depicted in Figure 8 reveals the crystals produced were prismatic or plate-like. This differs from the observations made by Zhang et al., that nanocrystals grown at low temperature are usually rod shaped [58]. Additional TEM images (Figures S5–S7) and a size distribution plot (Figure S8) have been included in the Supplementary Information.

#### 3.2.6. Cytotoxicity Studies

**Figure 9:**
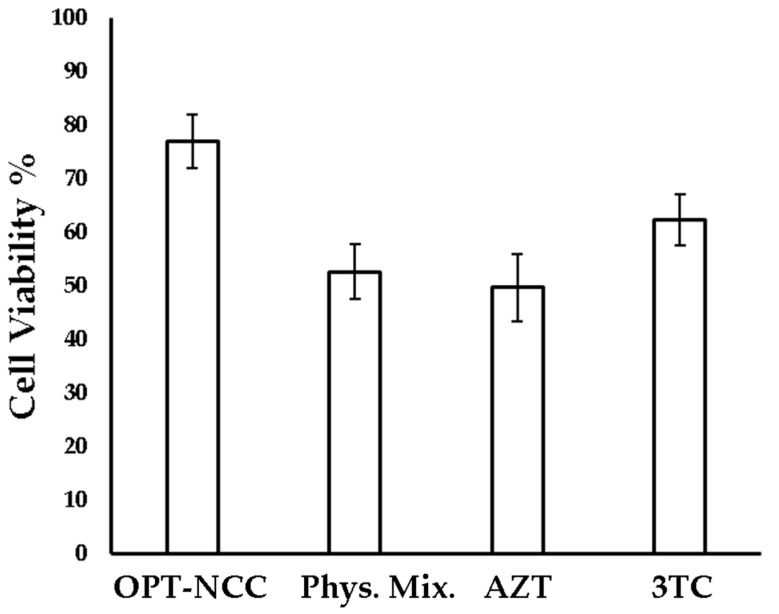
Cytotoxicity of NCC, individual API and physical mixture of active pharmaceutical ingredient (API).

The OPT-NCC exhibited improved cell viability when compared to that observed for the raw materials alone, which may be due to the stabilization induced by surfactants, and could have a shielding effect on the NCC. In addition, the presence of PEG, which forms the hydrophilic heads of TPGS 1000 may offer stealth properties to the NCC, minimizing uptake by HeLa cells [59,60]. Macrophages preferentially target negatively charged particles that are <1000 nm in size [61,62,63]. As the NCC produced in these studies are negatively charged, are <1000 nm in dimension and exhibit low HeLa cell toxicity, the NCC have the potential to target HIV harboring macrophages passively without affecting non-phagocytic cells. The summary of the in vitro cell viability is listed in Table 10 and depicted in Figure 9.

## 4. Conclusions

The formulation optimization for production of electrosterically stabilized 3TC and AZT NCC using a pseudo one-solvent bottom-up approach was successful. The PS and PDI were affected by the amount of surfactant used in the formulation. The presence of surfactants reduces the energy at the surface of nucleating crystals preventing crystal growth [64]. The PS was not as affected by the concentration of TPGS 1000 used than when SDS was used suggesting that electrostatic stabilisation was more effective in the synthesis of the NCC using a bottom-up approach. The PDI was significantly affected by the concentration of SDS used, due to the combined effect of increasing SDS and TPGS 1000 concentrations in the composition.

The ZP is primarily dependent on the presence of SDS and a linear relationship exists between SDS concentration and ZP. The data reveal that an increase in SDS content results in a proportional decrease in ZP for the NCC suspensions produced.

In order to achieve the development of a suitable technology for the delivery of AZT and 3TC, the optimization objective was to minimize the ZP, increase the stability of the formulation [65,66] and with particle size reduction, potentially target macrophages [61,62,63,67].

Characterisation of the OPT-NCC formulation using TEM revealed that the NCC were <500 nm. PXRD and DSC data indicate the OPT-NCC are crystalline, which could potentially increase stability, and co-crystal formation was confirmed using FTIR. SEM-EDX was used to determine the elemental surface composition of the NCC. The spectra confirmed the presence of stabilizer on the surface of crystals in addition to the presence of molecular peaks associated with the API.

In vitro cytotoxicity studies revealed the OPT-NCC were less cytotoxic to HeLa cells than the individual API and a physical mixture of the API. The presence of hydrophilic PEG heads in TPGS 1000 may be the reason for increased HeLa cell viability.

The use of DoE and cold sonoprecipitation to manufacture optimized NCC is an inexpensive, reproducible and precise method of manufacturing NCC with specific pre-defined desirable PS, PDI and ZP, while maintaining crystallinity of the individual compounds. The NCC produced in this study exhibit less toxicity to HeLa cells than individual components, which in turn, may result in reduced side effects of each API. The improvement of the API side effect profile may well improve patient adherence to therapy and lead to better treatment outcomes.

## Figures and Tables

**Table 1:** Summary of CCD experiments for nano co-crystals (NCC) optimization.

Std. Run	Form. Code	SDS % w/v	TPGS 1000 % w/v
4	1	1.00	2.00
11	2	0.50	1.50
5	3	0.00	1.50
2	4	1.00	1.00
7	5	0.50	0.79
8	6	0.50	2.21
13	7	0.50	1.50
6	8	1.21	1.50
1	9	0.00	1.00
3	10	0.00	2.00
9	11	0.50	1.50
10	12	0.50	1.50
12	13	0.50	1.50

Sodium dodecyl sulphate (SDS), α-tocopheryl polyethylene glycol succinate (TPGS 1000).

**Table 2:** Summary of optimized formulation conditions for the manufacture of optimized nano co-crystal (OPT-NCC).

Formulation Variable	Optimized Condition
**SDS concentration (X_1_)**	0.90% w/v
**TPGS 1000 concentration (X_2_)**	1.40% w/v

**Table 3:** Summary of variables used and responses for NCC produced using CCD.

Summary of CCD Experiments for Optimisation				Responses for NCC Produced Using CCD		
Std. Run	Form. Code	SDS % w/v	TPGS % w/v	PS nm	PDI	ZP mV
4	1	1.00	2.00	451.9 ± 43.1	0.689 ± 0.003	−45.0 ± 3.9
11	2	0.50	1.50	446.8 ± 52.8	0.478 ± 0.034	−27.7 ± 1.2
5	3	0.00	1.50	705.6 ± 92.3	0.474 ± 0.092	−22.3 ± 0.9
2	4	1.00	1.00	475.9 ± 22.3	0.506 ± 0.033	−30.5 ± 2.6
7	5	0.50	0.79	630.1 ± 73.2	0.414 ± 0.045	−27.1 ± 1.4
8	6	0.50	2.21	304.1 ± 38.6	0.498 ± 0.087	−21.9 ± 2.3
13	7	0.50	1.50	436.4 ± 67.1	0.471 ± 0.011	−25.9 ± 3.1
6	8	1.21	1.50	404.9 ± 73.6	0.462 ± 0.038	−37.0 ± 0.3
1	9	0.00	1.00	859.4 ± 101.2	0.448 ± 0.088	−13.3 ± 3.7
3	10	0.00	2.00	656.9 ± 77.2	0.284 ± 0.018	−13.5 ± 2.2
9	11	0.50	1.50	424.9 ± 21.7	0.432 ± 0.028	−26.2 ± 1.6
10	12	0.50	1.50	423.7 ± 13.2	0.449 ± 0.049	−25.3 ± 0.7
12	13	0.50	1.50	430.7 ± 52.1	0.466 ± 0.009	−24.8 ± 2.7

Shaded cells reflect results that have met the critical quality attributes (CQA) [25]. PS, particle size; PDI, polydispersity index; ZP, Zeta potential.

**Table 4:** ANOVA data for response surface quadratic model for PS.

Source	Sum of Squares	df	Mean Square	F-Value	*p*-Value Prob. > F
Model	2.71 *×* 10^5^	5	54,111.2	26.82	0.0002
A-SDS Conc.	1.68 *×* 10^5^	1	1.679 *×* 10^5^	83.23	<0.0001
B-TGPS Conc.	59,087.81	1	59,087.8	29.29	0.0010
AB	7965.56	1	7965.56	3.95	0.0873
A^2^	73,941.57	1	73,941.6	36.65	0.0005
B^2^	6594.86	1	6594.86	3.27	0.1135
Residual	14,121.22	7	2017.32		
Lack of Fit	13,763.08	3	4587.69	51.24	0.0012
Pure Error	358.14	4	89.54		
Corr. Total	2.85 *×* 10^5^	12			

Significant factors are reported in red.

**Table 5:** ANOVA data for response surface linear model for PDI.

Source	Sum of Squares	df	Mean Square	F-Value	*p*-Value Prob. > F
Model	0.061	3	0.02	6.37	0.0132
A-SDS Concentration	0.029	1	0.029	8.99	0.015
B-TGPS Concentration	2.37 × 10^−3^	1	2.37 × 10^−3^	0.74	0.412
AB	0.03	1	0.03	9.39	0.0135
Residual	0.029	9	3.21 × 10^−3^		
Lack of Fit	0.027	5	5.496 × 10^−3^	15.9	0.0096
Pure Error	1.38 × 10^−3^	4	3.457 × 10^−3^		
Corr. Total	0.09	12			

Significant factors are reported in red.

**Table 6:** ANOVA data for response surface linear model for ZP.

Source	Sum of Squares	df	Mean Square	F-Value	*p*-Value Prob. > F
Model	666.17	2	333.08	17.56	0.0005
A-SDS Concentration	659.42	1	659.42	34.76	0.0002
B-TGPS Concentration	6.75	1	6.75	0.36	0.5642
Residual	189.72	10	18.97		
Lack of Fit	184.85	6	30.81	25.32	0.0038
Pure Error	4.87	4	1.22		
Corr. Total	855.89	12			

Significant factors are reported in red.

**Table 7:** Formulation variables and associated responses.

Formulation Variables		Formulation Responses			Desirability
SDS % w/v	TPGS % w/v	PS Nm	PDI	ZP mV	
0.90	1.40	393.08	0.499	−33.47	1.000

**Table 8:** Summary of results for the NCC produced using optimum formulation parameters.

Response	Predicted Value	Experimental Value	% Predicted Error
**PS nm**	393.08	332.9 ± 42.85	18.08
**PDI**	0.499	0.474 ± 0.040	5.27
**ZP mV**	−33.47	−34.6 ± 5.56	3.27

**Table 9:** Summary of elemental analysis for the physical mixture, NCC-OPT and co-crystal.

Element	OPT-NCC	Uncoated NCC
	Atomic %	Atomic %
**C_k_**	56.97 ± 2.18	49.67 ± 1.21
**N_k_**	13.99 ± 0.94	20.81 ± 0.94
**O_k_**	25.87 ± 1.22	28.53 ± 1.02
**Na_k_**	0.26 ± 0.13	-
**S_k_**	2.97 ± 0.34	1.00 ± 0.03

**Table 10:** Summary of in vitro cytotoxicity results.

Compound	Cell Viability %	SD
OPT-NCC	76.9	5.0
Physical Mixture	52.6	5.1
AZT	49.6	6.2
3TC	62.3	4.7

## Supplementary Materials

The following are available online at https://www.mdpi.com/1999-4923/12/4/367/s1, Table S1: Summary of melting temperatures of AZT, 3TC, reported co-crystal value and the OPT-NCC. Figure S1: DSC thermogram of 3TC (Black), AZT (Orange) and OPT-NCC (Blue). Table S2: Summary of the comparison FTIR for the reported co-crystal and the OPT-NCC. Figure S2: A plot of the values reported in Table S2 reflecting a one-to-one agreement between the FTIR wavenumbers reported in the literature [68] (blue) and those recorded for the NCC (orange). Figure S3: FTIR spectra of the OPT-NCC (orange), 3TC (blue) and AZT (black). Figure S4: PXRD diffractograms of OPT-NCC and Co-crystal (Diffractogram obtained from CSD [69]). Figure S5: TEM of uncoated bottom-up micro co-crystal. Figure S6: TEM depicting the mean particle size of the OPT-NCC, Figure S7: TEM images of the smallest product obtained (OPT-NCC). Figure S8: DLS intensity size distribution curve of the OPT-NCC.
