# Supplementary material for: Quality by Design Optimization of Cold Sonochemical Synthesis of Zidovudine-Lamivudine Nanosuspensions
Source: Pharmaceutics. 2020 Apr 17;12(4):367. doi: 10.3390/pharmaceutics12040367 (PMC7238087; doi:10.3390/pharmaceutics12040367)
Supplement: Supplementary file 1 [file pharmaceutics-12-00367-s001.pdf]

pharmaceutics

MDPI

Supplementary Materials: Quality by Design Optimization of Cold Sonochemical Synthesis of Zidovudine-Lamivudine Nanosuspensions.

Bwalya A. Witika, Vincent J. Smith and Roderick B. Walker

1. Comparison of Nano co-crystal (OPT-NCC) and API

1.1 Differential Scanning Calorimetry

The melting point for 3TC and AZT are clearly visible with the presence of a sharp melting endotherm at 123.6 °C for AZT. 3TC exhibits a sharp melting endotherm with a melting point of 180.4 °C. The figure also depicts a thermogram in which the melting endotherm for the OPT-NCC occurs at 94.3 °C. The reported melting point of the co-crystal is 94–95 °C [1]. These data are summarised in Table S2.

Table 1. Summary of melting temperatures of AZT, 3TC, reported co-crystal value and the OPT-NCC.

Compound	AZT	3TC	Reported Co-Crystal [1].	OPT-NCC
Temp °C	123.6	180.4	94–95	94.3

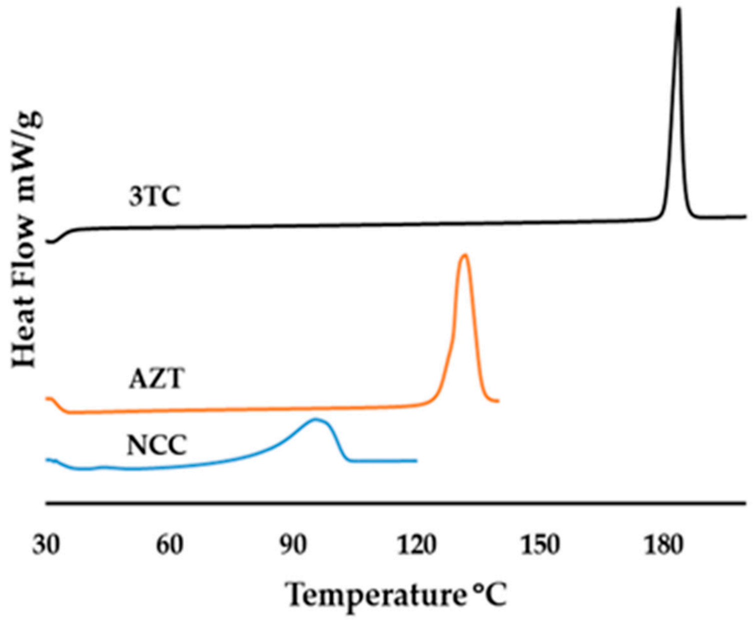

Figure S1. DSC thermogram of 3TC (Black), AZT (Orange) and OPT-NCC (Blue).

2. Comparison of Optimized Nano co-crystal (OPT-NCC) and the Reported Co-crystal

2.1. FTIR

The peaks are summarized and compared to those of the co-crystal reported in Table S2 and plotted in Figure S2 to confirm that the OPT-NCC has the identical molecular composition to that of the reported co-crystal.

Table S2. Summary of the comparison FTIR for the reported co-crystal and the OPT-NCC.

**FTIR**	
**Co-crystal Reported** [4]	**Co-crystal obtained Figure S2**
3532	3523
3414	3404
3310	3301
3219	3203
3087	3081
3014	-
2956	2948
2920	2915
2832	2816
2160	2156
2093	2088
1713	1707
1642	1630
1524	1523
1498	1493
1473	1469
1436	1431
1269	1265
1104	1101
1053	1049
827	826
782	779
758	759
741	740

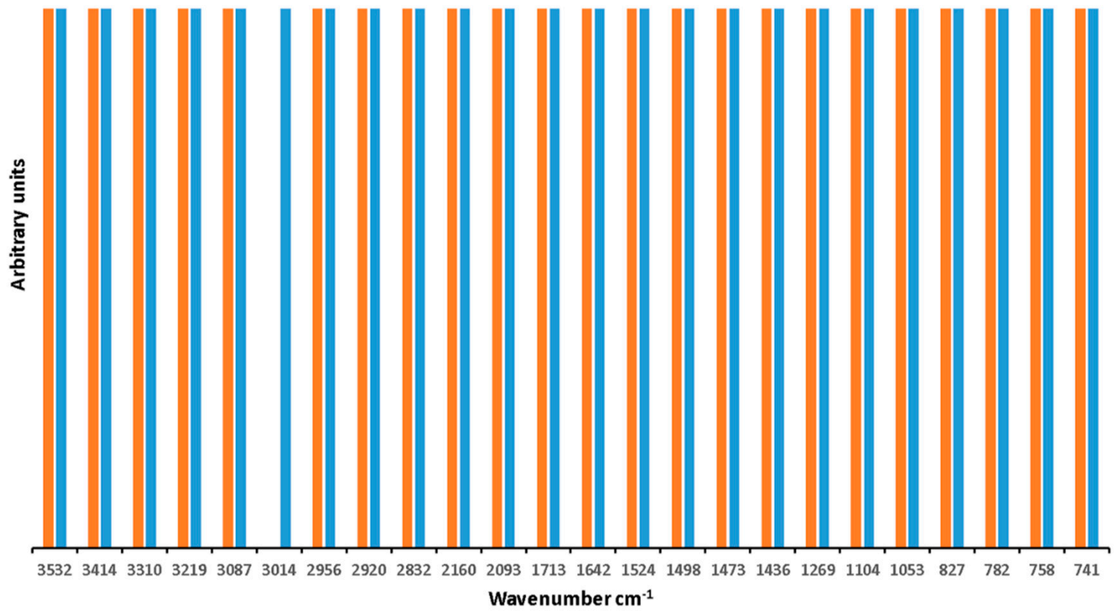

Figure S2. A plot of the values reported in Table S1 reflecting a one-to-one agreement between the FTIR wavenumbers reported in the literature [4] (blue) and those recorded for the NCC (orange).

The FTIR spectrum of the OPT-NCC is depicted in Figure S3. The FTIR spectrum of the co-crystal shows the presence of a peak at 3523 cm^{-1}. This peak is characteristic of presence of water molecules in the co-crystal [2,3].

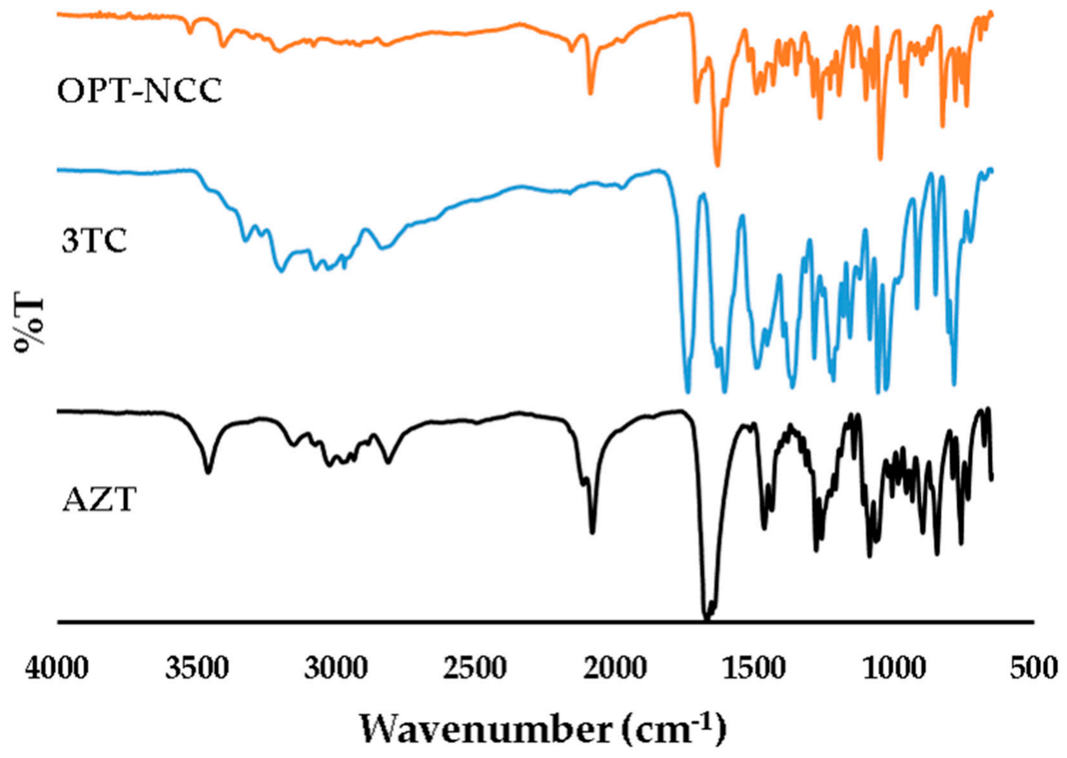

Figure S3. FTIR spectra of the OPT-NCC (orange), 3TC (blue) and AZT (black).

2.2 Powder X-Ray Diffraction

The powder diffractograms of the OPT-NCC and the reported co-crystal is depicted in Figure S4.The diffractograms have several overlapping peaks with different albeit with different intensities.

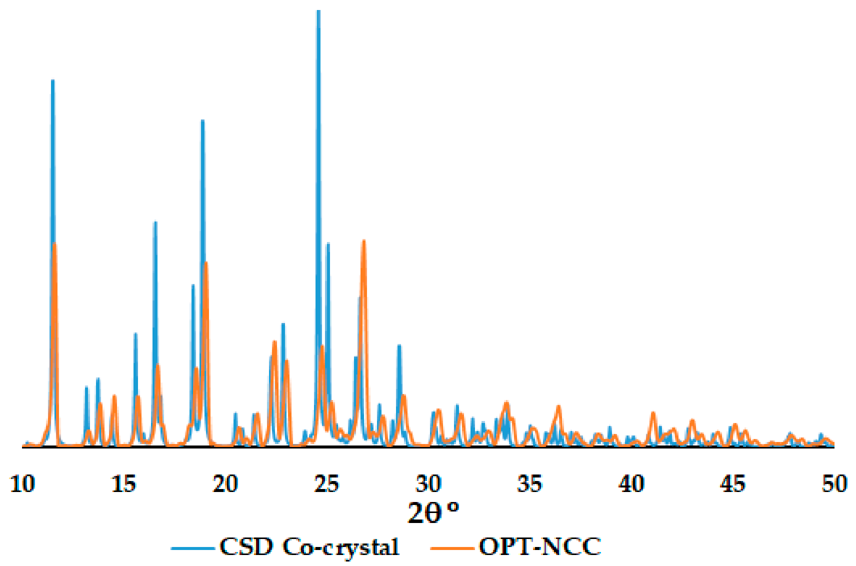

Figure S4. PXRD diffractograms of OPT-NCC and Co-crystal (Diffractogram obtained from CSD [4]). (refcode: WOMHEM).

3. Transmission Electron Microscopy

The micrographs depicted in figures S5, S6 and S7 reveal the particle size reduction associated with an increase in surfactant concentration. The uncoated micro co-crystals are depicted in figure S5 and are > 1 μm. The crystals depicted in Figure S6 have an average particle size of approximately 300 nm while in Figure S7 shows the smallest particles produced for the OPT-NCC with an average size of approximately 50 nm. Figure S8 depicts the particle size distribution by intensity for the OPT-NCC.

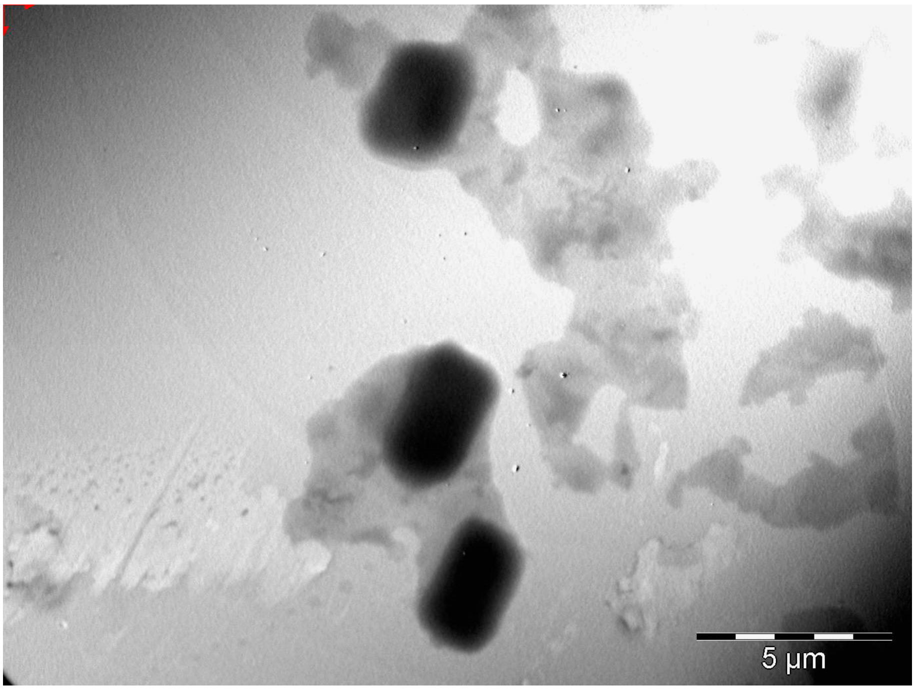

Figure S5. Uncoated bottom-up micro co-crystal.

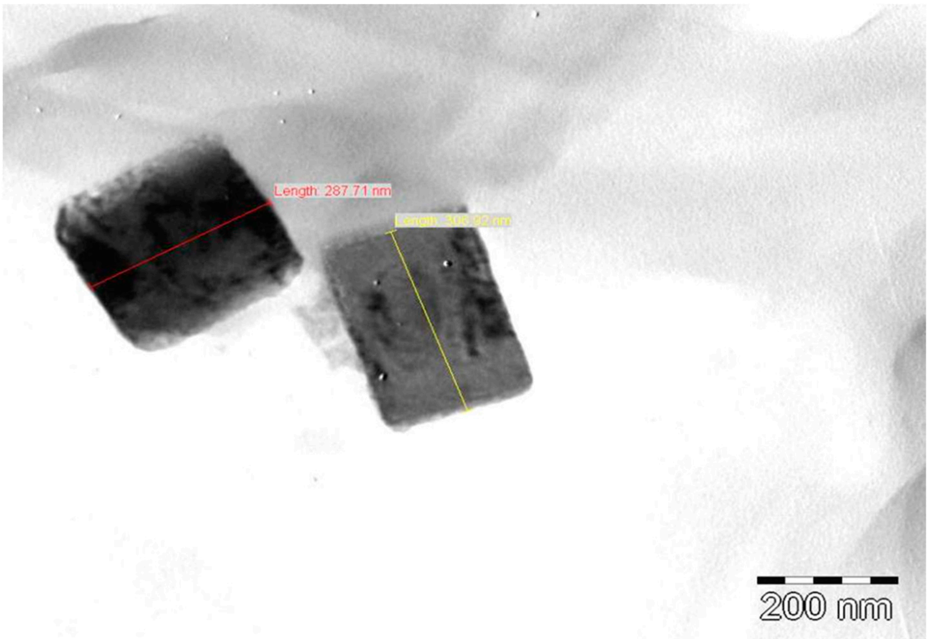

Figure S6. Micrograph depicting the mean particle size of the OPT-NCC.

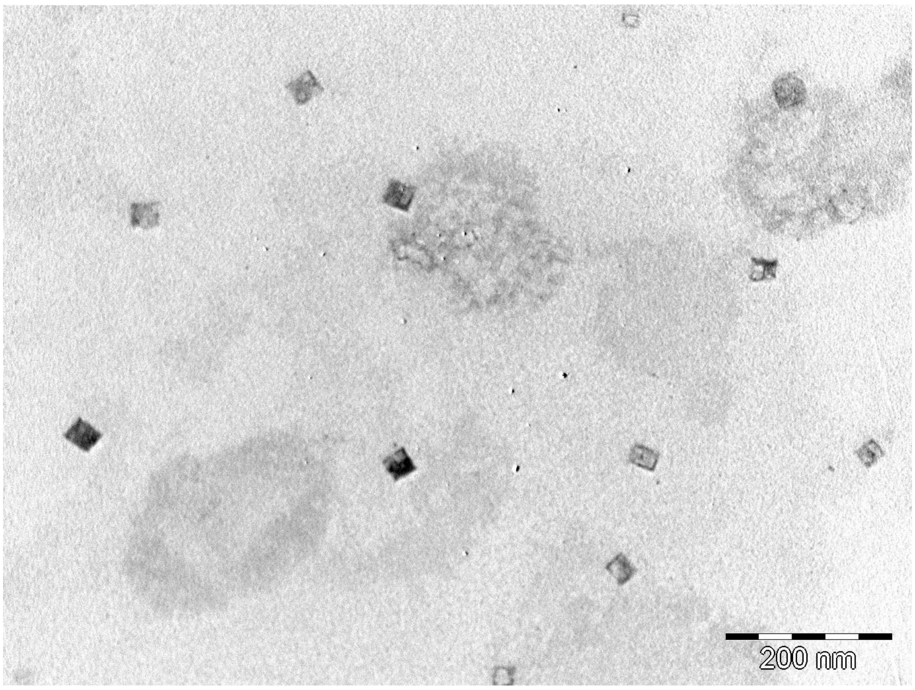

Figure S7. TEM images of the smallest product obtained (OPT-NCC).

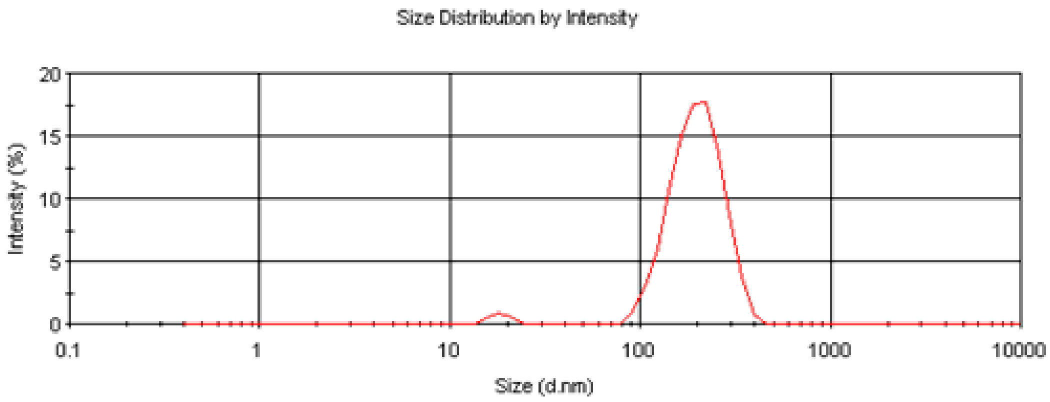

Figure S8. DLS intensity distribution curve of the OPT-NCC.

References

1. Lupin Limited WO 2009/116055 Al 2009.
2. Brink, G. Infrared Studies of Water in Crystalline Hydrates: $Ba(ClO_3)_2{\cdot}H_2O$. *Appl. Spectrosc.* **1976**, *30*, 630–631.
3. Falk, M.; Huang, C.-H.; Knop, O. Infrared Spectra of Water in Crystalline Hydrates: $KSnCl_3{\cdot}H_2O$, an Untypical Monohydrate. *Can. J. Chem.* **2006**, *52*, 2928–2931.
4. Groom, C.R.; Bruno, I.J.; Lightfoot, M.P.; Ward, S.C. The Cambridge structural database. *Acta Crystallogr. Sect. B Struct. Sci. Cryst. Eng. Mater.* **2016**, *72*, 171–179. refcode:WOMHEM.

© 2020 by the authors. Licensee MDPI, Basel, Switzerland. This article is an open access article distributed under the terms and conditions of the Creative Commons Attribution (CC BY) license (http://creativecommons.org/licenses/by/4.0/).
